# Supplementary material for: A Critical View of the Application of the APEX Software (Aqueous Photochemistry of Environmentally-Occurring Xenobiotics) to Predict Photoreaction Kinetics in Surface Freshwaters
Source: Molecules. 2019 Dec 18;25(1):9. doi: 10.3390/molecules25010009 (PMC7017383; doi:10.3390/molecules25010009)
Supplement: Supplementary file 1 [file molecules-25-00009-s001.zip › 3.2.4_gcc-4.4.0.zip!/3.2.4_gcc-4.4.0/3.2.4_gcc-4.4.0/doc/octave/html/interpreter/Quadratic-Programming.html]

Quadratic Programming - Untitled


Next: Nonlinear Programming,
Previous: Linear Programming,
Up: Optimization


---

### 24.2 Quadratic Programming

Octave can also solve Quadratic Programming problems, this is

```
     min 0.5 x'*H*x + x'*q
```

subject to

```
          A*x = b
          lb <= x <= ub
          A_lb <= A_in*x <= A_ub
```

— Function File: [x, obj, info, lambda] = **qp** (x0, H, q, A, b, lb, ub, A\_lb, A\_in, A\_ub)  
> Solve the quadratic program
>
> ```
>                min 0.5 x'*H*x + x'*q
>                 x
> ```
>
> subject to
>
> ```
>                A*x = b
>                lb <= x <= ub
>                A_lb <= A_in*x <= A_ub
> ```
>
> using a null-space active-set method.
>
> Any bound (A, b, lb, ub, A\_lb,
> A\_ub) may be set to the empty matrix (`[]`) if not
> present. If the initial guess is feasible the algorithm is faster.
>
> The value info is a structure with the following fields:
>
> `solveiter`: The number of iterations required to find the solution. `info`: An integer indicating the status of the solution, as follows: 0: The problem is feasible and convex. Global solution found. 1: The problem is not convex. Local solution found. 2: The problem is not convex and unbounded. 3: Maximum number of iterations reached. 6: The problem is infeasible.
